# Comment on Paditz et al. The Pharmacokinetics, Dosage, Preparation Forms, and Efficacy of Orally Administered Melatonin for Non-Organic Sleep Disorders in Autism Spectrum Disorder During Childhood and Adolescence: A Systematic Review. *Children* 2025, *12*, 648

**DOI:** 10.3390/children12101361

**Published:** 2025-10-09

**Authors:** Paul Gringras, Beth A. Malow, Carmen M. Schröder

**Affiliations:** 1 Department of Women and Children’s Health, King’s College London, London SE5 9RS, UK; 2 Sleep Disorders Division, Department of Neurology, Vanderbilt University Medical Center, Quechee, VT 05059, USA; beth.malow@vumc.org; 3 Department of Child and Adolescent Psychiatry, Strasbourg University Hospital, 67000 Strasbourg, France; schroderc@unistra.fr; 4 CNRS UPR 3212, Sleep Clock Light and Neuropsychiatry, Institute for Cellular and Integrative Neurosciences, 67084 Strasbourg, France; 5 Faculty of Medicine, Strasbourg University, 67081 Strasbourg, France; 6 Department of Psychiatry, Mental Health and Addictology, Strasbourg University Hospital, 67000 Strasbourg, France

We are writing in response to the recently published article, “The Pharmacokinetics, Dosage, Preparation Forms, and Efficacy of Orally Administered Melatonin for Non-Organic Sleep Disorders in Autism Spectrum Disorder During Childhood and Adolescence: A Systematic Review” by Paditz et al. 2025 [1].

While we recognise the topic’s importance, we wish to raise serious concerns regarding the review’s methodology, study selection, and interpretation of findings. Drawing on our clinical and academic expertise and a thorough review of the relevant literature, we find that the article contains several inaccuracies, inconsistencies, exclusions of pertinent data, and unsupported assertions. These issues risk misrepresenting the current evidence base and may lead to inappropriate treatment recommendations for insomnia in children and adolescents with autism spectrum disorder (ASD).

## 1. Inconsistent Application of Inclusion Criteria

The review states that included studies must either exclusively involve participants with ASD or report subgroup analyses for ASD populations. However, four of the five included studies [2,3,4,5] enrolled participants with multiple comorbidities and provided limited or no ASD-specific subgroup analyses. Only Hayashi et al. [4] included subpopulation analyses, and these were based on variables such as sex, age, weight, sleep onset latency (SOL) at baseline, prior ramelteon use, intellectual disabilities, ADHD, and height—not ASD-specific outcomes. This undermines the consistency and rigour of the stated inclusion criteria. Only Cortesi et al. [6] adhered more closely to the criteria, though it focused on a controlled-release formulation, limiting its relevance to the review’s conclusions regarding immediate-release melatonin.

## 2. Exclusion of Key Studies and Misrepresentation of Existing Evidence

Several high-quality, widely cited RCTs—such as Gringras et al. (2012), Appleton et al. (2012), and Gringras et al. (2017) [7,8,9]—were excluded without explanation, despite meeting inclusion criteria and being referenced in authoritative guidelines. [10,11].

Gringras et al. (2012), an NIHR-funded, dose-ranging RCT of immediate-release melatonin in 146 children with neurodevelopmental disorders (including 60 with autism), found that 18% responded to a 0.5 mg dose, while others required higher doses (12.38% needed 12 mg) [7]. Thirty participants with autism were assigned to each arm (melatonin and placebo), with no significant difference in response compared to participants without autism. Although the study showed that immediate release (IR) melatonin reduced sleep latency, total sleep time increased less than expected. This was because children receiving melatonin woke, on average, 30 min earlier than those in the placebo group. The authors hypothesised that evening exogenous melatonin gradually advances the sleep phase—leading to earlier sleep onset and earlier wake time, thus limiting overall sleep duration. This finding was the impetus to study a prolonged-release formulation, using similar rigorous methodology in Gringras et al. (2017) [9]. The 2012 paper explicitly noted that only head-to-head trials comparing immediate-release, prolonged-release melatonin, and other sedatives would help clinicians and families determine the safest and most effective option.

The claim that Gringras et al. (2017) [9] lacks subgroup analysis is demonstrably incorrect. The study includes subgroup analyses based on ADHD status, age, and geographic region. Criticism of its screening and randomisation is also inconsistent, given that other included studies reported similar or higher dropout rates or omitted such details entirely.

Furthermore, concerns about funding sources and data analysis responsibilities are selectively applied. For example, Hayashi et al. (2022), an industry-sponsored study, is not scrutinised to the same extent [4]. This selective application of quality assessment criteria raises concerns about bias.

## 3. Omission of Publicly Available Pharmacokinetic Data for Children

The review states—both in the abstract and on page 6—that pharmacokinetic data for sustained-release melatonin in children with ASD are unavailable. This is demonstrably false. Publicly accessible data are available in EMA assessment reports for the paediatric prolonged-release melatonin product (Slenyto), including detailed pharmacokinetic and pharmacodynamic data presented in Tables 4 and 5 and Figures 5–7 of the document (https://www.ema.europa.eu/en/documents/variation-report/circadin-h-c-695-p46-0019-epar-assessment-report_en.pdf accessed on 25 June 2025) and on page 29 of the public assessment report (https://www.ema.europa.eu/en/documents/assessment-report/slenyto-epar-public-assessment-report_en.pdf accessed on 25 June 2025).

In addition, these data are included in Schroder et al. (2021)—a peer-reviewed paper cited by the authors themselves [12]. The omission of this directly relevant and easily accessible data is both puzzling and concerning.

## 4. Misleading Claims About Dose–Response Data

The review omits publicly available pharmacokinetic data for paediatric prolonged-release melatonin, despite this information being accessible via EMA and other regulatory bodies. These data, as well as those published in Schroder et al. (2021), were not only available but also cited by the authors [12].

Assertions that dose–response relationships have only been studied in observational cohorts of nine children are incorrect. Controlled trials such as Gringras et al. (2012) and Gringras et al. (2017) provide dose–response data, and relevant pharmacokinetics are available both in regulatory reports and published studies [7,9].

## 5. Clinical Relevance and Generalisability

The review does not address clinically meaningful outcomes such as changes in sleep onset latency, total sleep time, behavioural impacts, or parent-reported quality of life—all of which are well-documented in excluded trials e.g., Schroder et al. 2019; Malow et al. 2021 [13,14].

It is notable that the authors also fail to reference recent consensus work, such as the International Pediatric Sleep Association-led international expert melatonin review [11], despite one of the authors (Ipsiroglu) having co-authored the publication. This publication does cover the use of melatonin for children with ASD, and suggests a dose range of 2–10 mg. In keeping with Gringras 2012, [7] this paper also comments that “Clinical trials may be needed to consider which formulation, IR vs. PR is more appropriate for insomnia in a specific population”.

## 6. Inaccurate Statements Regarding Regulatory Guidance and Recommendations

The review incorrectly claims that no dosing guidance exists for melatonin in children. In fact, such recommendations are available in the approved Summary of Product Characteristics (SmPC) and have been published in algorithmic form [12]. Similarly, the claim that no RCTs have examined prolonged-release melatonin in children with ASD is contradicted by multiple peer-reviewed studies discussed above and regulatory approvals (EMA, TGA, SwissMedic).

## 7. Use of Non-Standard Terminology and Limited Clinical Insight

The use of terms such as “delayed-release” and “non-delayed” is non-standard and may confuse readers. Accepted terminology is “prolonged-release” and “immediate-release”. The review also conflates delayed sleep phase disorder (DSPD) with sleep difficulties in ASD, failing to note that the latter often involves early and mid-sleep awakenings—a distinct clinical profile.

## 8. Conclusions

We submit these comments in the interest of maintaining scientific rigour and transparency in reviews that inform clinical practice. The exclusion of key studies, inconsistent application of quality criteria, and selective interpretation of evidence undermine the utility of this review for clinicians and researchers. We recommend that the article be substantially amended or accompanied by a published correction or commentary to address these concerns, prevent misinterpretation of the evidence base, and prevent non-evidence-based clinical practice.
